# Statistical Optimization and Machine-Learning-Based Analysis of Palm Oil Pretreatment (3–90% FFA) for Enhanced Free Fatty Acid Conversion in Biodiesel Production

**DOI:** 10.1021/acsomega.5c07792

**Published:** 2025-10-13

**Authors:** Maythee Saisriyoot, Kulthawat Tepjun, Anusith Thanapimmetha, Sakaophat Wibunlaksanakun, Suphitchayanee Namboonlue, Tunyaboon Laemthong, Penjit Srinophakun

**Affiliations:** † Department of Chemical Engineering, Faculty of Engineering, 54775 Kasetsart University, Bangkok 10900, Thailand; ‡ Department of Chemical Engineering, Faculty of Engineering, Thammasat School of Engineering, 649975 Thammasat University, Pathum Thani 12120, Thailand

## Abstract

Palm oil is a highly efficient feedstock for large-scale biodiesel production, as it yields significantly more oil per agricultural area than other common oil crops, such as soybeans or sunflowers. However, crude palm oil often faces a high level of free fatty acid (FFA) problems, hindering biodiesel production. Pretreatment, such as esterification, is thus employed to convert FFA to fatty acid methyl ester (FAME) and avoid undesired byproducts. Response surface method (RSM) has been widely and effectively used to optimize the pretreatment conditions. To address this challenge, raw palm oil (a mixture of palm stearin and palm fatty acid distillate) with the initial FFA of 3–90% was used in the pretreatment process. A four-factor-three-level Box–Behnken experimental design was deployed to estimate the final FFA as a function of reaction time (0.5–4 h, X_1_), molar ratio of methanol to FFA (3:1–24:1, X_2_), catalyst (0.5–8 wt % based on FFA, X_3_), and initial FFA (3–90%, X_4_). Three different mathematical models were obtained and validated over different ranges of FFA in palm oil. The optimum conditions were 2.73 (X_1_), 22.02:1 (X_2_), 3.90 (X_3_), and 21.88 (X_4_) for 3–30% FFA; 2.34 (X_1_), 16.57:1 (X_2_), 3.12 (X_3_), and 60.00 (X_4_) for 30–60% FFA; and 2.40 (X_1_), 16.05:1 (X_2_), 3.12 (X_3_), and 90.00 (X_4_) for 60–90% FFA, respectively. After validation, the results showed that palm oil at 1–30 and 60–90% FFA gave fewer errors of 0.60 and 0.58% respectively, than the other ranges of 30–60% at 1.25%. Therefore, a machine learning approach was used to improve the optimum conditions, comparing decision tree, random forest, and gradient boosting. It was found that the decision tree gave the highest *R* ^2^ of 0.9762, RMSE of 1.2130, and MAE of 0.4070. The new optimum conditions from the predictive model of 3–90% FFA were 2.25 (X_1_), 15:1 (X_2_), 11.5 (X_3_), and 46.5 (X_4_) via a gradient boosting model with the least percentage error to obtain %final FFA less than 1%.

## Graphical abstract

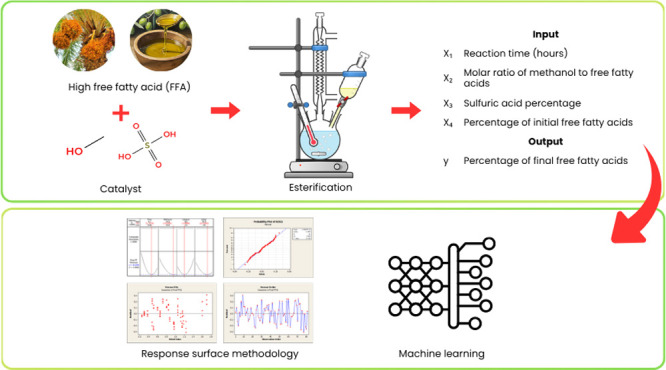

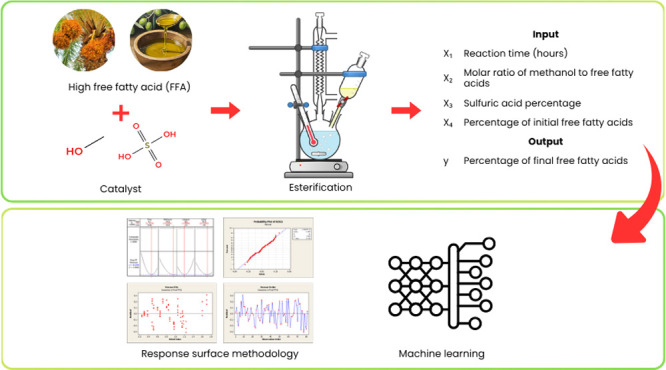

## Introduction

The increasing global energy demand and the current global warming issue have led to the development of alternative energy sources and their associated production processes.^1,2^ Biodiesel, a renewable and biogenic fuel, has emerged as a promising alternative to conventional fossil diesel, offering significant environmental benefits, including reduced greenhouse gas emissions and lower particulate matter.^3,4^ Biodiesel is a mixture of fatty acid methyl esters (FAME) produced through transesterification. The procedure requires alcohols (typically methanol or ethanol) and a catalyst (a strong base like NaOH or KOH).^5,6^ The reaction that converts oil to biodiesel is simplified in eq 1. Biodiesel properties and stability rely on the chemical structure of the feedstock.^7^ Among various feedstocks, palm oil stands out globally as a significant source for biodiesel production due to its high oil yield per unit area compared to other oil crops.^8^ Palm oil provides advantages over other oil crops regarding yield, scalability, and economic viability.^9^ However, to avoid the food-feed-fuel dilemma, low-quality palm oil with high free fatty acids (FFA) is considered for energy, such as whole seed palm oil (mainly from community palm oil extraction) and palm fatty acid distillate (from the palm oil refinery process). The initial FFA for biodiesel production should be less than 1% for the high-yield transesterification and two-step process.^10–12^ A pretreatment step, especially acid esterification as in eq 2, to remove FFA, is crucial and is often performed on these feedstocks to avoid the saponification, which lowers biodiesel yield and complicates the biodiesel separation process.^12,13^ Therefore, pretreatment is required for palm oil raw material or any waste oils that contain FFA of more than 1%.

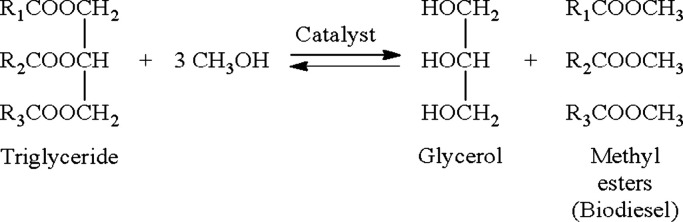

1

$$R-\mathrm{COOH}+R-\mathrm{OH}\overset{\mathrm{Catalyst}}{\to }R-\mathrm{COOR}+H_{2}O$$ 2

The biodiesel production cost is influenced by several factors, such as feedstock cost, chemicals, utilities, etc.^14^ Over 85% of the operating cost typically comes from the raw materials and utilities.^8^ However, in the case of waste cooking oil, the cost of pretreatment, accounting for approximately 15% of the total cost, is required.^15^ Thus, optimized conditions for feedstock pretreatment are required for biodiesel production, leading to the overall sustainability.^16,17^ Procedures like the Response Surface Methodology (RSM) have been studied extensively for optimized conditions. RSM is a collection of mathematical and statistical techniques specifically designed to model and analyze problems where a response of interest is influenced by several independent variables.^18^ Its primary objective is to optimize this response, whether it is to maximize yield, minimize cost, or achieve a desired quality characteristic.^19^ RSM is highly effective because it identifies the optimal operating conditions and provides insights into the intricate interplay and synergistic or antagonistic effects of the various input variables.^20,21^ Through biodiesel production, RSM was deployed to determine the Kusum biodiesel production with the molar ratio of methanol to oil of 7.5:1, with 0.95% (w/w) KOH at 63.3 °C for 1.54 h.^22^ The optimized biodiesel production process, using waste corn oil and rapeseed oil with the use of synthesized nanocatalyst, was obtained from RSM with a Box-Behnken experimental design, where its molar ratio of methanol to oil of approximately 13:1, with 8% (w/v) at 65 °C for 7 h.^23^ Moreover, RSM was also used in the optimization of diesel engine performance and emissions from biodiesel–diesel blends and provided less than 10% error in prediction.^24^

While RSM is a valuable tool, its ability to describe complex and nonlinear relationships among process variables during FFA removal in the esterification step can be limited. Therefore, this study explores the application of Machine Learning (ML), a powerful data-centric approach, to identify complex patterns for more accurate prediction and process optimization. A significant advantage of ML is that models can be trained on historical or experimental data without requiring extensive theoretical knowledge of process mechanisms. Furthermore, these models can capture multivariate effects and complex relationships, often resulting in greater accuracy and robustness. ML is broadly divided into supervised, unsupervised, and reinforcement learning.^25^ This study employs a supervised learning approach, using labeled data for training and final FFA prediction. The methods selected for this study include traditional regression techniques (linear and polynomial with degree of 2) and more advanced tree-based ensemble methods, which are particularly well-suited for capturing nonlinear interactions without requiring predefined relationships between variables. The evaluated models are the Decision Tree (DT), which classifies and predicts using a hierarchical structure;^26^ the Random Forest (RF), which combines multiple DT to reduce overfitting and improve accuracy;^27^ and the Gradient Boosting (GB), which sequentially corrects errors to enhance predictions.^28^

This comparative analysis is expected to identify the most accurate optimization for biodiesel production by applying both RSB and ML to the esterification-based pretreatment of palm oil. Thus, the optimization focused on key parameters: reaction time, methanol-to-FFA molar ratio, sulfuric acid percentage, and initial FFA, particularly relevant when using low-quality oil. The targeted final free fatty acid after the treatment was 1%. For the RSM, the initial FFA was divided into three ranges (3–30, 30–60, and 60–90%) with polynomial equations proposed, while ML was applied to improve the *R* ^2^ of RSM (polynomial regression) prediction.

## Materials and Methods

### Chemicals

Palm stearin (PS, initial FFA of 10%) and palm fatty acid distillate (PFAD, initial FFA of 90%) were purchased from Patum Vegetable Oil Company Limited (Pathum Thani, Thailand). Potassium hydroxide (Ajax, Finechem, Sydney, NSW, Australia), sulfuric acid (Qrec reagent, Auckland, New Zealand), methanol (99.99 wt %, Carlo Erba Reagent Company Limited, Italy), and 2-propanol (99.99 wt %, Carlo Erba Reagent Company Limited, Italy) were of analytical grade.

### Design of Experiment

A Box-Behnken experimental design (Minitab Software) was deployed to predict the most influential factor affecting the overall biodiesel production cost, reaction time (X_1_), methanol to fatty acid molar ratio (X_2_), %catalyst loading (X_3_), and %initial FFA (X_4_). The minimum of 3% and maximum of 90% FFA was investigated in this study by mixing PS and PFAD. The parameters and their ranges were independently separated into 3 sets according to the percentage of initial FFA of the raw material, shown in Table 1, namely 3–30, 30–60, and 60–90%. Three ranges of investigation were adopted from Khine.^29^

**Table 1:** Parameter and Its Level for Use in Predictive Model Construction

		parameter level		
parameter	symbol	–1	0	+1
Set 1:3–30% FFA Palm Oil				
reaction time (h)	X_1_	0.5	2.25	4.0
methanol:palm oil	X_2_	10:1	17:1	24:1
% (w/v) catalyst	X_3_	1.0	4.5	8.0
% initial FFA	X_4_	3.0	16.5	30.0
Set 2:30–60% FFA Palm Oil				
reaction time (h)	X_1_	0.5	2.25	4.0
methanol:palm oil	X_2_	3.0	11.5	20.0
% (w/v) catalyst	X_3_	1.0	3.5	6.0
% initial FFA	X_4_	30.0	45.0	60.0
Set 3:60–90% FFA Palm Oil				
reaction time (h)	X_1_	0.5	2.25	4.0
methanol:palm oil	X_2_	3.0	11.5	20.0
% (w/v) catalyst	X_3_	0.5	2.25	4.0
% initial FFA	X_4_	60.0	75.0	90.0

### Esterification-FFA Removal

Palm oil with different percentages of FFA (parameter level of −1, 0, and +1) according to the experimental design in Table 1 (3, 16.5, 30, 45, 60, 75, and 90% by weight) was made using the combination of PS and PFAD as mentioned earlier. The procedure was adapted from Laemthong et al.^30^ A 100 g oil mixture was poured into a round-bottomed flask equipped with a reflux condenser and heated to a reaction temperature of 60 °C. The solution of H_2_SO_4_ (based on the weight percent of FFA in oil as shown in eq 3) in methanol (eq 3), prepared using a statistical experimental design, was also added to the flask. The mixture was stirred at the same speed of 500 rpm for all runs. Once the reaction was complete, 20% v/v water at 40 °C was added to the mixture. The mixture was then placed in a separation funnel to separate the water. Finally, the mixture was heated at 105 °C for 30 min. The treated palm oil was analyzed for %FFA using eq 5.

### Calculation of the Amount of Reactants for the Esterification Reaction

The amount of methanol and sulfuric acid used in each run is calculated by eqs 3–5, respectively, where 32 is the molecular weight of methanol.

$$H_{2}{\mathrm{SO}}_{4}\;\mathrm{used}\;\mathrm{in}\;\mathrm{reaction}\;(g)=\frac{\%\mathrm{Cat}\times \%{\mathrm{Ini}}_{\mathrm{FFA}}\times M_{{\mathrm{oil}}_{\mathrm{react}}}}{100}$$ 3

$$\mathrm{Methanol}\;\mathrm{used}\;\mathrm{in}\;\mathrm{reaction}\;(g)=\frac{32\times {\mathrm{Met}}_{\mathrm{ratio}}\times {\mathrm{Ini}}_{\mathrm{FFA}}\times M_{{\mathrm{oil}}_{\mathrm{react}}}}{M_{w_{\mathrm{FFA}}}}$$ 4

$$\%\mathrm{FFA}=\frac{N_{\mathrm{KOH}}\times V_{\mathrm{KOH}}\times 270.002\times 100}{1000\times W_{\mathrm{sample}}}$$ 5

where Met_ratio_ is the methanol-to-FFA molar ratio; *M*_oilreact_ is the weight of oil used in the esterification reaction (approximately 50 g); *M*_wFFA_ is the molecular weight of FFA (270.002 g/mol); %Cat is the percentage amount of catalyst (wt % based on FFA); %Ini_FFA_ is the percentage amount of initial FFA (wt %); *N*_KOH_ is KOH concentration (M); *V*_KOH_ is KOH volume (mL); *W*_sample_ is sample weight (g).

The average molecular weights of FFA and triglyceride were analyzed by Gas Chromatography (Shimadzu, model 2010). The calculated molecular weight of FFA is 270.002 g/mol, and the molecular weight of triglyceride is 848.006 g/mol.

### FFA Quantification

The major property of palm oil after esterification was quantified as the FFA to analyze the removal percentage. The procedure was modified from previous studies.^7,30^ The biodiesel sample of 5 g was mixed with 50 mL of 2-propanol. The mixture was then titrated with 0.05–0.5 M KOH using phenolphthalein as an indicator. The FFA was then calculated using eq 5.

### Process Optimization via Response Surface Methodology (RSM)

The response surface procedure analyzed the experimental data to fit the following second-order polynomial model (eq 6), as follows, predicted for optimization of both esterification and transesterification of a mixture of PFAD and PS;

$$Y=\beta_{ko}+\sum_{i=1}^{3}\beta_{ki}x_{i}+\sum_{i=1}^{3}\beta_{kii}x_{i}^{2}+\sum_{i=1}^{2}\sum_{j=i+1}^{3}\beta_{kij}x_{i}x_{j}$$ 6

where *Y* was the response (% conversion), β_*ko*_, β_*ki*_, β_*kii*_, and β_*kij*_ were constant coefficients, and *x_i_* was the uncoded independent variable. This ridge-maximal option was used to compute the estimated ridge of maximum response for increasing radii from the center of the origin design.

The empirical mathematical model was tested using analysis of variance (ANOVA) at a 95% confidence level. The ANOVA was used to check the significance of the second-order models. *F*-value determined the statistical significance of the second-order equation (eqs 7–11). In general, the calculated *F*-value is used to reject the null hypothesis, where all the regression coefficients are zero. The calculated *F*-value is defined as the ratio between the mean square of regression (MSR) and the mean square of error (MSE), where MSR and MSE are obtained by dividing the sum of squares (SSR) and the sum of residual (SSE) by the respective degrees of freedom (df). Meanwhile, tabulated *F*-values are obtained from the *F*-distribution based on (df) for regression and residuals, respectively, at a specified level of significance, which is defined as the α value.^31^

$$Y=\beta_{0}+\beta_{1}x+\varepsilon$$ 7

$$Y=\beta_{0}+\beta_{1}x+\beta_{2}x^{2}+\varepsilon$$ 8

where β_0_, β_1_, and β_2_ are coefficients; ε is an error.

To determine the efficiency of the regression equation, the coefficient of determination, *R* ^2^ was used. A higher *R* ^2^ is preferred for higher accuracy of the prediction. *R* ^2^ relates to SST and SSE as in eq 9. However, as *R* ^2^ is quite sensitive, *R*_adj_^2^ is used for decision-making. Therefore, *R* ^2^ and *R*_adj_^2^ were used together to verify the regression models.

$$R^{2}=\frac{\left(\mathrm{SST}-\mathrm{SSE}\right)}{\mathrm{SST}}$$ 9

$$R_{\mathrm{adj}}^{2}=1-\frac{\mathrm{MSE}}{\mathrm{MST}}$$ 10

$$\mathrm{MST}=\frac{\mathrm{SST}}{n-1}$$ 11

### Machine Learning Procedure

The data set used in this study was derived from previously conducted esterification experiments. All computations and algorithm implementations were performed using Python (with libraries such as pandas and numpy) and scikit-learn. The data set comprises four independent variables—reaction time (h), molar ratio of alcohol to fatty acid, sulfuric acid concentration (%), and initial FFA content—while the dependent variable of interest is the final FFA after esterification. The data were subsequently split into training (80%) and testing (20%) sets using train_test_split with a random_state of 42 to enable reproducibility of results. This study trained and evaluated five regression models: Decision Tree (DT), a nonparametric tree-based method; Random Forest (RF), an ensemble of decision trees that reduces overfitting; Linear Regression (LR), which assumes a linear relationship between predictors and response; Gradient Boosting (GB), a boosting algorithm that sequentially improves accuracy; and Polynomial Regression (degree = 2), which extends linear regression with polynomial terms to capture nonlinear interactions.

### Accuracy Assessment

The models’ results, as well as their performance in predicting the final percentage of FFA, were evaluated using three metrics: Root Mean Squared Error (RMSE), *R*-squared (*R* ^2^), and Mean Absolute Error (MAE). Root Mean Squared Error (RMSE) quantifies the average magnitude of prediction errors and is defined as

RMSE=1n∑i=1n(yi−yi^)2 12

where *y*_*i*_ is the observed value, *ŷ*_*i*_ is the predicted value, and *n* is the number of observations. A lower RMSE value indicates better model performance in predicting sugar concentration.

*R*-squared (*R* ^2^) measures the proportion of variance in the dependent variable that the independent variables can explain as

R2=1−∑i=1n(yi−yi^)2∑i=1n(yi−yi¯)2 13

where *y̅* is the mean of the observed values. This metric indicates how well the model’s predictions approximate the actual data, with a value closer to 1 representing better predictive performance.

Mean Absolute Error (MAE) calculates the average absolute differences between predicted and observed values, offering a straightforward measure of prediction accuracy:

MAE=1n∑i=1n|yi−yi^| 14

This metric shows how close the model’s predictions are to the actual values, with lower MAE values indicating better performance.

## Results and Discussion

### Analysis of FFA Removal Using 3–30% FFA Feedstock

For 3–30% FFA feedstock, the optimized reaction time was found to be 2.73 h, with the ratio of methanol to FFA of 22.02, and the amount of catalyst is 3.90% by weight. The optimized composition of FFA was 21.82% with a final composition of FFA of 0.126% by weight, as shown in Figure 1. A normality test was conducted using Ryan–Joiner (RJ), indicating a normal distribution of residual data with a *P*-value greater than 0.1. This demonstrates that residual data has no significant effect. The variance stability of the residual data set was further analyzed using the Levene test with its *F*_w_ less than *F*_α,*k*–1,*N*–*k*_. The results also provide a nonstatistical difference in the residual plot. The equal distribution of the residual data set was also observed. This indicates the stability of the variance as seen in Figure 1. Furthermore, as per Krushal–Wallis method, the *P*-value is greater than 0.05, with *F*_H_ being greater than *X*_0.05,80_^2^(101.879) identified as independently distributed data. The pattern of data distribution was unpredictable (Figure 1). Thus, from the prior data analysis, the analysis and variance of FFA removal was conducted using the MINITAB program with a 95% confidence level, with degrees of freedom of 80. The results are summarized in Table 2. The relationship between each parameter is described by eq 15. The critical value of the *F*-distribution was greater than the standard value; thus, the model is valid.

$$\begin{array}{cc} Y & =5.08093-1.17801X_{1}-0.20438X_{2}-0.38691X_{3}-0.02997X_{4}+0.13460X_{1}^{2}+0.00342X_{2}^{2}\\ & +0.03002X_{3}^{2}+0.00107X_{4}^{2}+0.01833X_{1}X_{2}+0.04033X_{1}X_{3}-0.00556X_{1}X_{4}+0.00150X_{2}X_{3}\\ & -0.00014X_{2}X_{4}+0.00043X_{3}X_{4} \end{array}$$ 15

where *X*_1_: reaction time (h), *X*_2_: mole ratio of methanol to FFA, *X*_3_: sulfuric acid percentage, *X*_4_: percentage of initial FFA.

**Table 2:** ANOVA of the Esterification Process of 3–30% FFA by Weight^a^

source	DF	SS	MS	*F*	*P*-value
regression (model)	14	12.0745	0.86246	34	0.000
residual error	66	1.6741	0.02536		
total	80	13.7486			

a *R* ^2^ = 87.82%; *R* ^2^(adj) = 85.24%.

**Figure 1:**
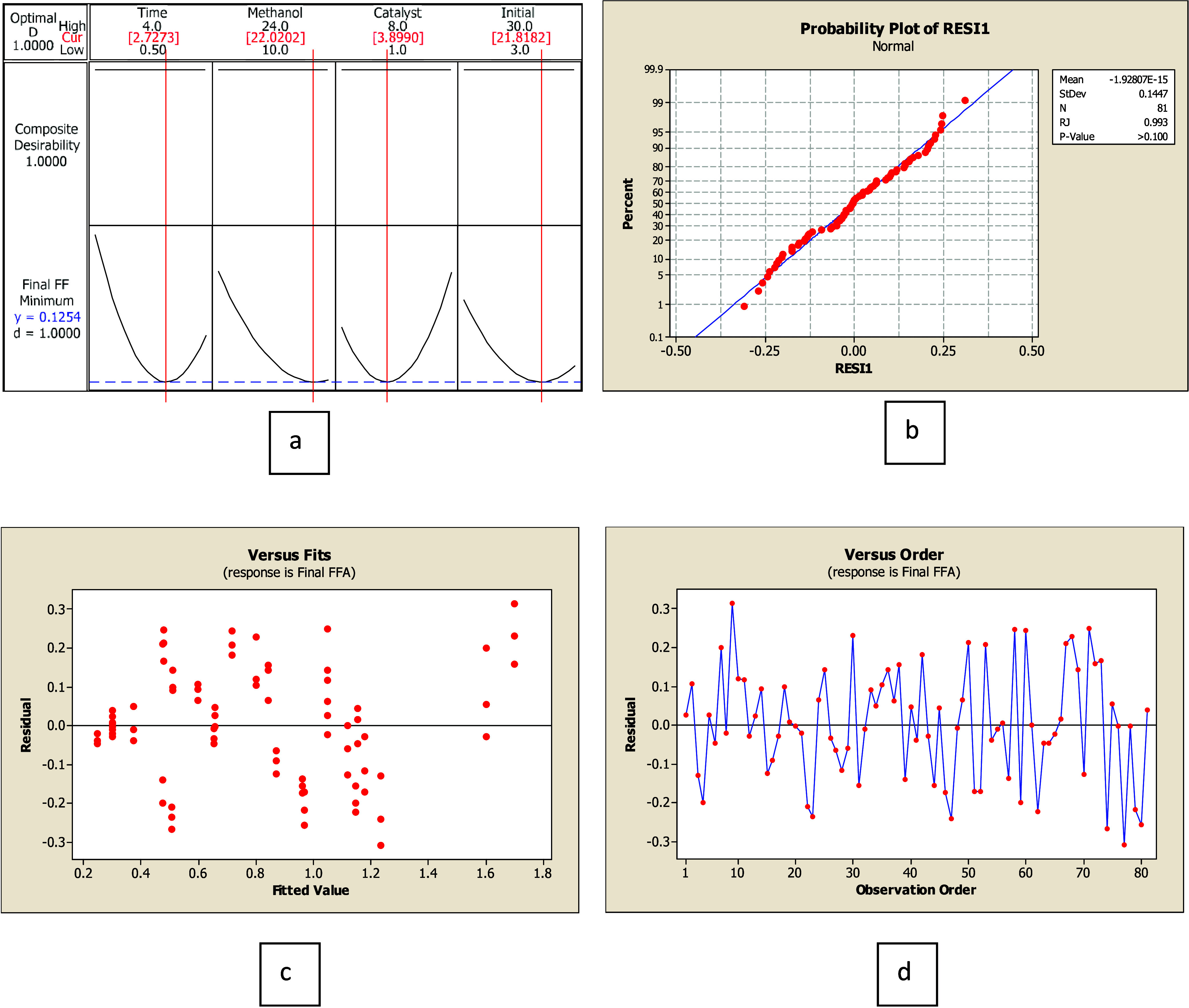
Optimization of 3–30% FFA by weight of feedstock: (a) Optimized condition, (b) residual data set distribution, (c) residual data set comparison to the model, and (d) residual data set comparison to observation order.

### Analysis of Free-Fatty Acid Removal Using 30–60% FFA Feedstock

Similarly, the Box–Behnken design was used to predict the residual FFA in the pretreated palm oil. A total of 81 experimental runs were conducted to construct a predictive model. The optimized conditions for these feedstocks were found to be 2.34 h, with a molar ratio of methanol to FFA of 16.57:1, using 4.28% catalyst weight, as shown in Figure 2. The RJ statistical analysis method was deployed to validate the model. The *P*-value was found to be 0.058, which is higher than the critical value of 0.05, indicating that the residual data follow a normal distribution, as shown in Figure 2 and Table 3. In addition, the variance stability of the Levene test (*F*_w_ of 1.85) was less than its standard of 3.11. The *P*-value at 95% confidence was higher than 0.05. This indicates the constant variance of residual data. However, the distribution was found to be a pattern, suggesting it was not evenly distributed. However, the predictive model could be used since the other statistical values were within the acceptable range. The ANOVA model was constructed based on the experimental data with 95% confidence with degrees of freedom of 80. The predictive model of parameter interaction was described in eq 16. The ANOVA of the model yielded an *R* ^2^ value of 96.05, with a *P*-value less than 0.05. The result indicates the model was valid.

$$\begin{array}{cc} Y & =22.1755-5.8829X_{1}-2.0889X_{2}-2.4237X_{3}-0.2379X_{4}+0.4462X_{1}^{2}+0.0572X_{2}^{2}\\ & +0.0723X_{3}^{2}-0.0010X_{4}^{2}+0.1536X_{1}X_{2}+0.3446X_{1}X_{3}-0.0036X_{1}X_{4}+0.0774X_{2}X_{3}\\ & -0.0082X_{2}X_{4}+0.00035X_{4} \end{array}$$ 16

where *X*_1_: reaction time (h), *X*_2_: mole ratio of methanol to free fatty acids, *X*_3_: sulfuric acid percentage, *X*_4_: percentage of initial FFA.

**Table 3:** ANOVA of the Esterification Process of 30–60% FFA by Weight^a^

source	DF	SS	MS	*F*	*P*-value
regression (model)	14	1463.28	104.520	114.70	0.000
residual error	66	60.14	0.911		
total	80	1532.42			

a *R* ^2^ = 96.05%; *R* ^2^(adj) = 95.21%.

**Figure 2:**
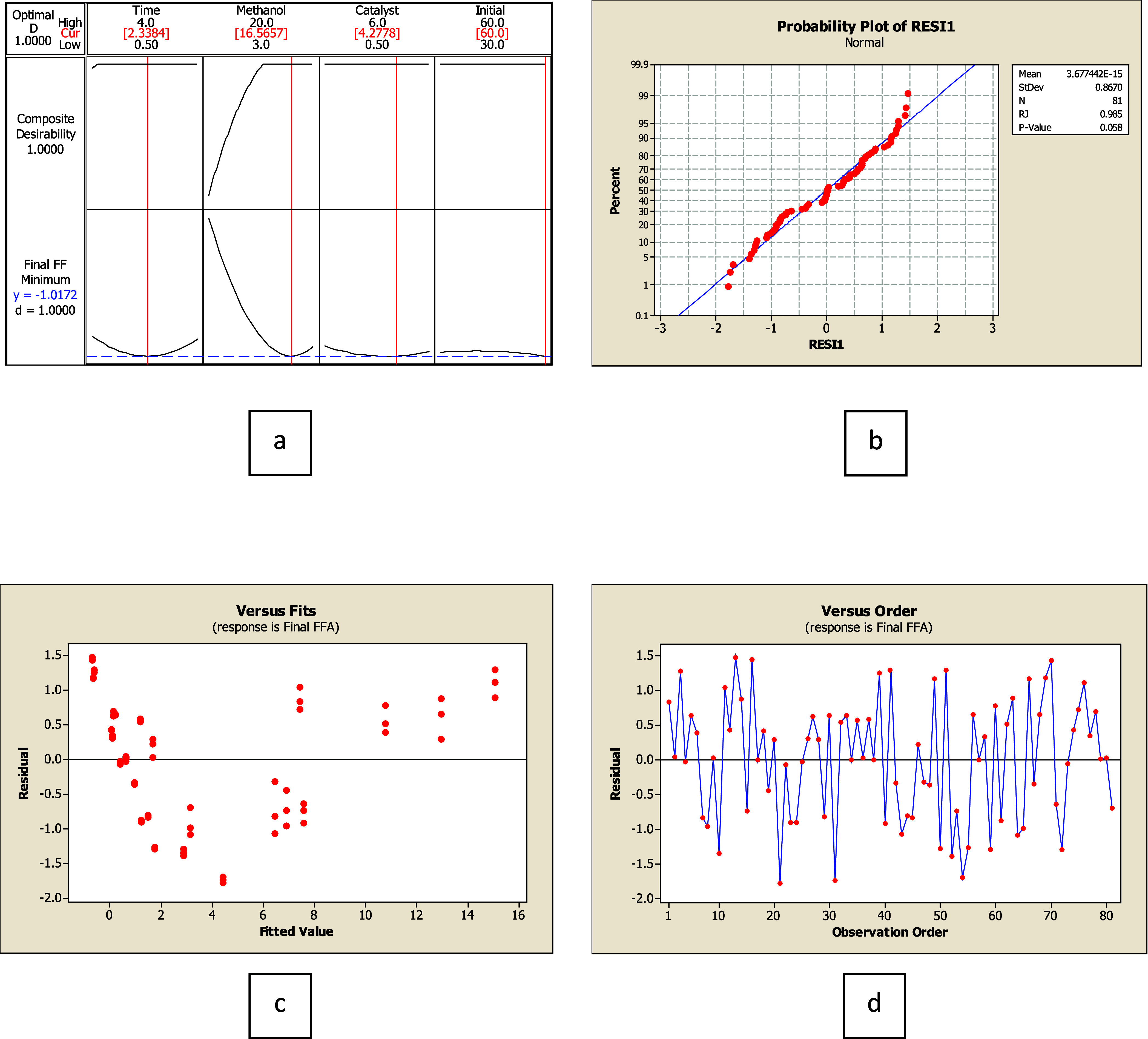
Optimization of 30–60% FFA by weight of feedstock: (a) Optimized condition, (b) residual data set distribution, (c) residual data set comparison to the model, and (d) residual data set comparison to observation order. Analysis of FFA removal using 60–90% FFA feedstock.

### Analysis of FFA Removal Using 60–90% FFA Feedstock

According to ANOVA, the optimized condition of 60–90% FFA feedstock was found to be 2.41 h of reaction time, the molar ratio of methanol to FFA of 16.05:1, with 3.12% catalyst by weight, and the initial FFA amount of 90%. A normal distribution was tested via RJ on the residual data to validate the model. The results provide a *P*-value of more than 0.1 with an *R*-squared value of 0.99 at 95% confidence. The residual data set has a normal distribution as shown in Figure 3. Meanwhile, its variance stability (*F*_w_ of 0.00) was less than *F*_0.05,2,78_ with a *P*-value of 0.883, indicating that the variance stability was constant with a pattern. Thus, a relationship between all parameters was analyzed using the MINITAB program. The predictive model of parameter interaction was described in eq 17. The ANOVA of the model exhibited *R* ^2^ of 97.82, with a *P*-value of 0.00, which is less than 0.05. The result indicates the model was valid. All statistical parameters for the models are summarized in Table 4.

$$\begin{array}{cc} Y & =47.1682-12.6843X_{1}-4.3381X_{2}-8.0205X_{3}+0.3184X_{4}+0.8012X_{1}^{2}+0.1292X_{2}^{2}\\ & +0.7833X_{3}^{2}-0.0001X_{4}^{2}+0.2752X_{1}X_{2}+1.1266X_{1}X_{3}+0.0103X_{1}X_{4}+0.2683X_{2}X_{3}\\ & -0.0144X_{2}X_{4}-0.0417X_{3}X_{4} \end{array}$$ 17

where *X*_1_: reaction time (h), *X*_2_: mole ratio of methanol to free fatty acids, *X*_3_: sulfuric acid percentage, *X*_4_: percentage of initial free fatty acids.

**Figure 3:**
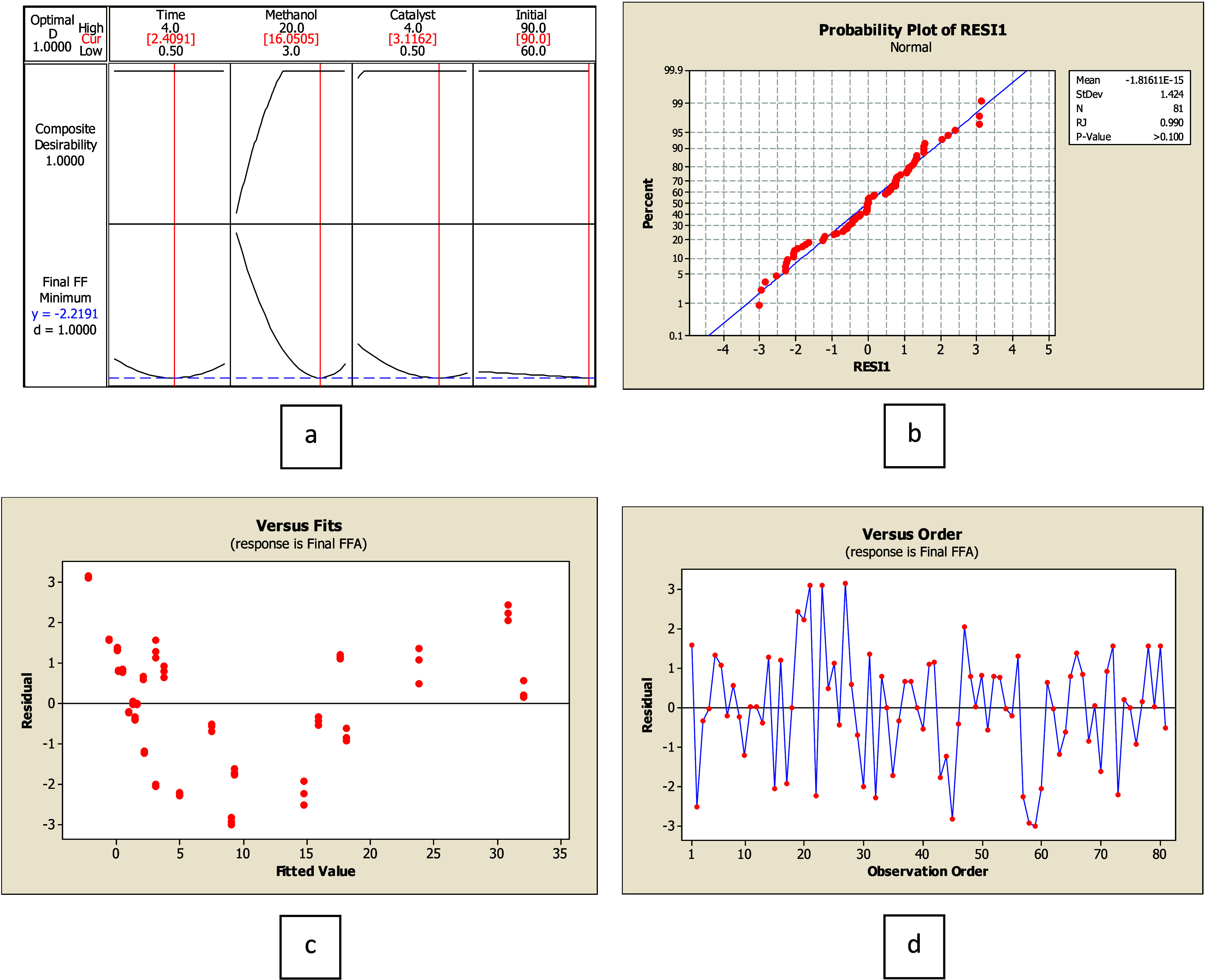
Optimization of 60–90% FFA by weight of feedstock: (a) Optimized condition, (b) residual data set distribution, (c) residual data set comparison to the model, and (d) residual data set comparison to observation order.

**Table 4:** ANOVA of the Esterification Process of 60–90% FFA by Weight^a^

source	DF	SS	MS	*F*	*P*-value
regression (model)	14	7291.50	7291.50	211.77	0.000
residual error	66	162.32	2.46		
total	80	7453.82			

a *R* ^2^ = 97.82%; *R* ^2^(adj) = 97.36%.

### Response Surface Methodology Validation

To validate the model, actual experiments were conducted using the optimized conditions for the esterification process for each feedstock (sets 1–3). Using the constructed three models, the differences between the prediction and experimental data fell below 10%, within the acceptable range (Table 5). The difference percentage was calculated in eq 13. However, these three models contain two connecting points, which are 30 and 60% FFA by weight. A MATLAB code was built by limiting the final FFA to 1%. Afterward, each condition was experimentally performed to compare and select the condition (Table 5). The validation showed that the RSM prediction models of 3–30 and 60–90% FFA fit well with the experimental data and seem to be promising for use in the future.

%Error=(PredictedValue−ActualValue/ActualValue)×100 18

**Table 5:** Response Surface Methodology Validation of Each Constructed Equation by Comparing with Actual Experimental Data

%FFA model	parameter				experimental data of the final FFA after esterification (% by weight)	%FFA conversion		% error
	*X*_1_	*X*_2_	*X*_3_	*X*_4_		prediction	actual	
3–30	2.73	22:1	3.80	21.88	0.1254	96.66	96.08	0.60
30–60	0.5	20:1	4.81	34.33	0.59	97.06	98.29	1.25
60–90	4	20:1	0.69	88.49	1.50	98.87	98.30	0.58

To improve the optimization, Machine Learning was employed for experimental data to improve the RSM model. Instead of using three different models for suitable ranges of %initial FFA, experimental data sets were analyzed to indicate the influence of parameters. To minimize the %error between the model and the experimental data, different models were used.

### Integrating a Machine Learning Approach to Optimize the Esterification Condition Instead of Using Three Different Models

#### Model Performance

From the performance evaluation using key statistical metrics presented in Table 6, the Decision Tree model demonstrated the highest predictive accuracy for Final FFA, with the highest *R* ^2^ of 0.9762, indicating that it explains nearly all (97.62%) of the variation in Final FFA. Additionally, it achieved the lowest RMSE (1.2130) and MAE (0.4070), indicating that its predictions are, on average, the closest to the actual values.

**Table 6:** Statistical Values of Each Model Based on Experimental Data (3–90% Initial FFA)

model	*R* ^2^	RMSE	MAE
linear regression	0.4181	6.0001	3.9929
polynomial regression (*n* = 2)	0.7514	3.9220	2.4749
decision tree	0.9762	1.2130	0.4070
random forest	0.9389	1.9451	0.6267
gradient boosting	0.9495	1.7681	0.6420

The other tree-based models, Gradient Boosting and Random Forest, also performed strongly, with *R* ^2^ values of 0.9495 and 0.9389, respectively, alongside low RMSE (1.7681 and 1.9451), demonstrating their robustness and high predictive ability. In contrast, Linear Regression performed the worst, with an *R* ^2^ of 0.4181, a high RMSE of 6.0001, and a large MAE of 3.9929, indicating its limited ability to capture the relationships within the data. Polynomial Regression (*n* = 2) performed somewhat better than Linear Regression (*R* ^2^ = 0.7514; RMSE = 3.9220) but fell significantly short of the tree-based models’ performance.

Overall, the Decision Tree was the best model for the Final FFA prediction, followed by Gradient Boosting and Random Forest. However, a limitation of tree-based models is their lack of an explicit mathematical equation, which makes interpreting the effects of individual input variables more challenging, despite their strong predictive performance.

#### Residual Plot

Residual plots are recognized as an essential tool for diagnosing model validity, a standard practice in applied machine learning.^32^ In these plots, residual values are plotted against their corresponding predicted values. For an appropriate model, the residuals are expected to be randomly scattered around the zero line, exhibiting no discernible pattern. Such a distribution indicates that the model has fully captured the relationships within the data and that no systematic bias is present.^33^ From the analysis of the residual plots for all five models (Figure 4), a clear distinction was observed between the regression-based and tree-based model groups.

**Figure 4:**
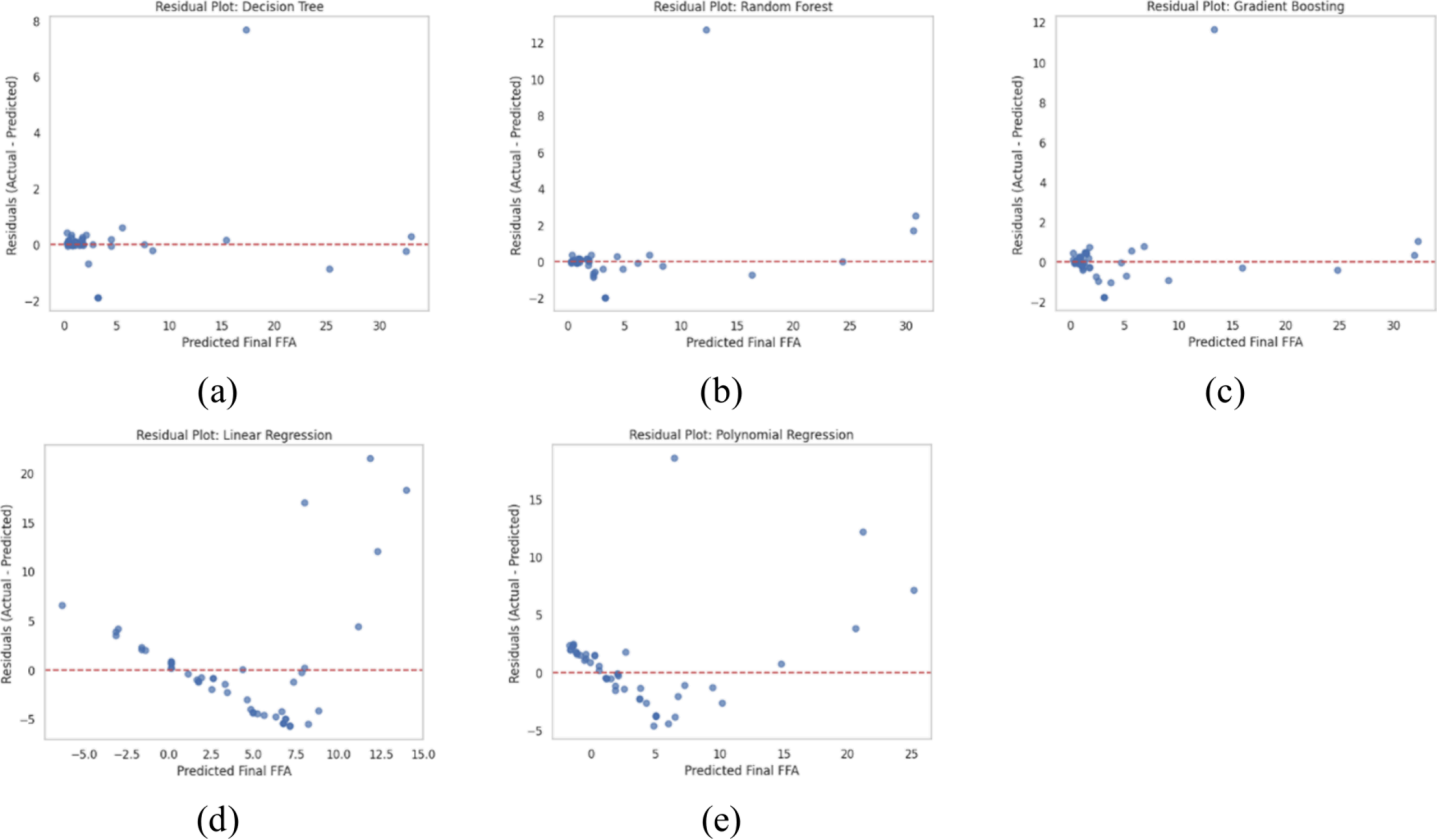
Analysis of the residual plot of each model.

A nonrandom pattern was exhibited in the residual plots for the Linear Regression and Polynomial Regression (*n* = 2) models. The data points were clustered, indicating that the complex and nonlinear relationships between the process variables and the final FFA were not fully captured. As a result, systematic errors were introduced in the predictions made by these models. In contrast, the tree-based models, namely Decision Tree, Random Forest, and Gradient Boosting, demonstrated superior results. The residual plots for these models, particularly for Random Forest and Gradient Boosting, showed a distinct random distribution of residuals around the zero line, with no evident pattern being observed (Figure 5). This observation indicates that the tree-based models achieved a higher degree of flexibility and efficiency in understanding and predicting the esterification process in biodiesel production. This finding is consistent with other recent studies in the field.^34^

**Figure 5:**
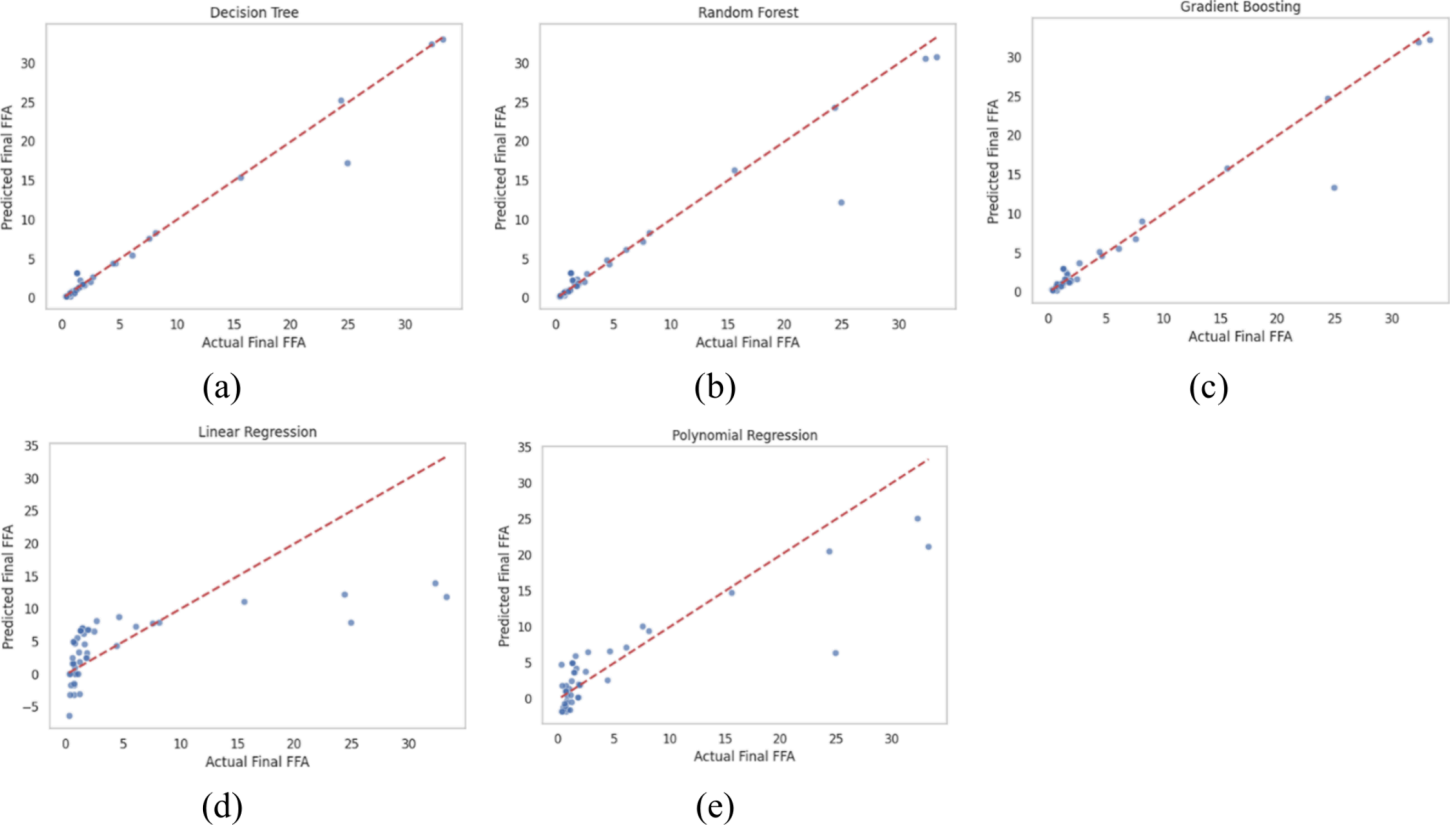
Comparison among different predictive models via a machine learning method: (a) decision tree, (b) random forest (RF), (c) gradient boosting (GB), (d) linear regression (LR), and (e) polynomial regression (PR).

#### Actual vs Prediction

Similarly, Figure 5 compares the predicted and actual values of Final FFA, tree-based models (including Decision Tree, Random Forest, and Gradient Boosting) outperform regression models in predictive performance. The data points predicted by tree-based models are closely distributed along the 45-degree line, indicating that the predicted values strongly agree with the actual values from the experiments. This demonstrates the ability of tree-based models to capture complex and nonlinear relationships within the data set effectively.

This observation is further supported by the performance metrics presented in Table 6, where the Decision Tree model shows the highest performance with an *R* ^2^ of 0.9762 and the lowest RMSE of 1.2130, followed by Gradient Boosting with an *R* ^2^ of 0.9495 and RMSE of 1.7681, and Random Forest with an *R* ^2^ of 0.9389 and RMSE of 1.9451.

In contrast, regression models exhibit a clustering of predicted points in some areas and poor dispersion overall, with many points deviating from the 45-degree line, reflecting their limited ability to describe complex relationships within the process. The Polynomial Regression (*n* = 2) model, a nonlinear form, performs less effectively than the tree-based models, with an *R* ^2^ of 0.7514 and an RMSE of 3.9220. In contrast, Linear Regression shows the poorest performance, with an *R* ^2^ of only 0.4181 and an RMSE as high as 6.0001. This comparison demonstrates that tree-based models, particularly Decision Trees, are significantly more appropriate and effective for Final FFA prediction. These models can be subsequently used to control and optimize the production process more efficiently.

Comparing the percentage error of each model, gradient boosting provides the least %error. The error of a total of 3–90% was not different from the least error of the separated models. To obtain an all-in-one model, gradient boosting is a suitable approach for optimizing the reaction conditions of palm oil containing 3–90% FFA. Meanwhile, the RSM approach requires 3 different models for FFA removal. However, the accuracy of predictive models relies heavily on the number of experimental data. The new predictive model of 3–90% FFA was 2.25 (*X*_1_), 15:1 (*X*_2_), 11.5 (*X*_3_), and 46.5 (*X*_4_) via a gradient boosting model with the least percentage error to obtain %final FFA less than 1%.

#### Correlations

From the Pearson correlation analysis between the independent variables (features) and the dependent variable (Final FFA), as illustrated in the correlation heatmap (Figure 6), each variable exhibits a distinct degree of association with Final FFA. The methanol to FFA molar ratio (mol_ratio) displayed the strongest and most significant negative correlation (*r* = −0.62), indicating that the Final FFA tends to diminish as the molar ratio increases. This observation is consistent with the principle of transesterification, where adding a greater amount of methanol drives the reaction forward, thereby reducing the residual FFA in the product.^35^

**Figure 6:**
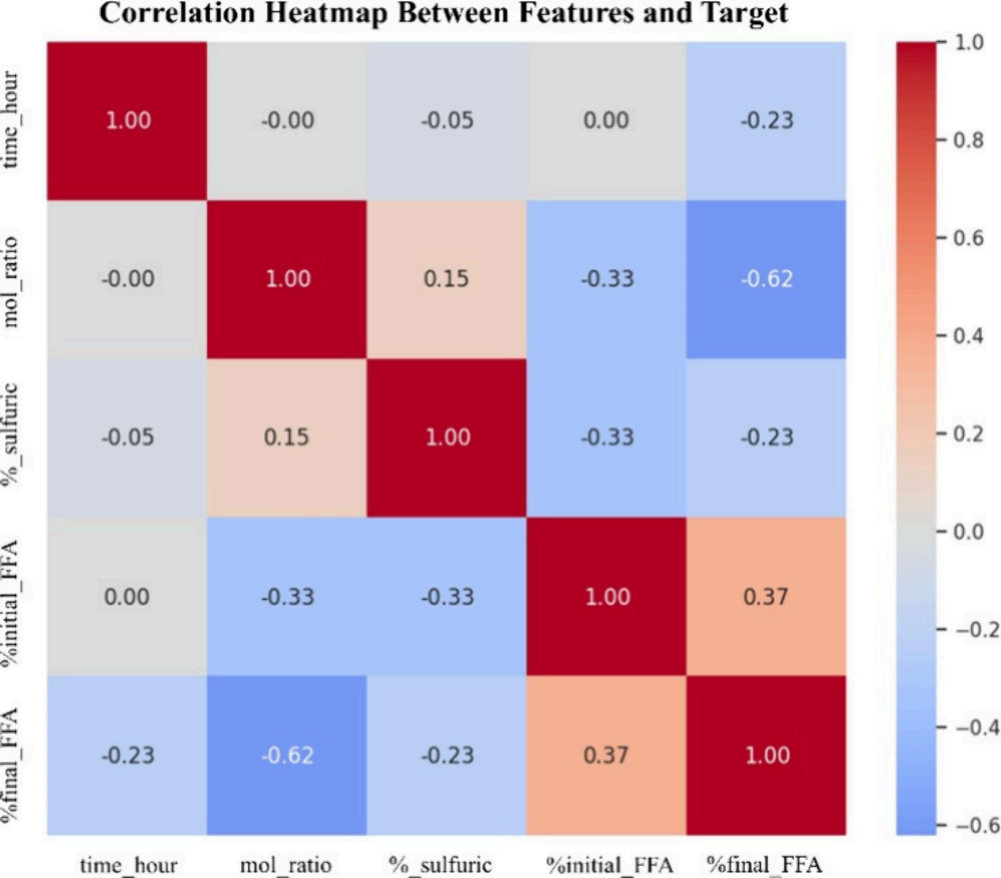
Correlation heatmap between experimental parameters affecting the targeted FFA removal.

Additionally, reaction time (time_hour) and sulfuric acid concentration (%_sulfuric) were also negatively correlated with Final FFA (*r* = −0.23 for both), suggesting that extending reaction time or adding acid catalyst contributes to a slight reduction in FFA, in agreement with the reaction mechanisms.^36^ In contrast, the %initial FFA content (%initial_FFA) was positively correlated with %final FFA (*r* = +0.37), implying that higher initial FFA levels are more likely to result in greater residual FFA after the reaction, even when process conditions are optimized.^37^ This correlation analysis is crucial for understanding the influence of process variables on biodiesel production. It can aid in selecting key parameters for further process optimization and improved predictive model performance.

## Conclusions

Even though palm oil is considered a promising feedstock for large-scale biodiesel production, high FFA in the feedstock ultimately leads to lower biodiesel yield. Chemical pretreatment, known as esterification, is normally employed to remove excess FFA to achieve high conversion to biodiesel. Process optimization is eventually required, especially in very large-scale production, where tons of palm oil feedstock are processed on a daily basis. Small improvements in conversion or yield can be significant, which can affect the overall production cost. With the conventional techniques like the response surface model, three different mathematical models are obtained and validated through experimental data for the optimized conditions. With our proposed mathematical models, the optimal conditions of the esterification process are available for use in high-volume production. The experimental data indicate a high accuracy of the models, with percentage differences considered acceptable when compared to the models. However, by implementing a machine learning method on the experimental data exhibits trends and the influence of each parameter. The increase in the molar ratio of methanol to oil resulted in better FFA removal. Meanwhile, other parameters such as reaction time or the amount of catalyst added to the reaction slightly impact the FFA removal process. The quality of the feedstock, as demonstrated by the percentages of initial FFA, is a significant factor limiting the biodiesel yield. RSM models offer a higher molar ratio of methanol to FFA but a smaller amount of catalyst employed in the process, which is appropriate for instrumental safety, particularly in cases of corrosion. Nevertheless, running the process at a high reaction volume could use more energy than doing it at a lower one. This element needs to be further investigated with the industrial restrictions in order to achieve maximum optimization for scale-up. Moving forward, considering the transport phenomena insight for a large-scale production would be another angle for process improvement.
